# Metabolic Profiles of Whole, Parotid and Submandibular/Sublingual Saliva

**DOI:** 10.3390/metabo10080318

**Published:** 2020-08-06

**Authors:** Marco Meleti, Eleonora Quartieri, Rita Antonelli, Margherita E. Pezzi, Benedetta Ghezzi, Maria Vittoria Viani, Giacomo Setti, Emanuela Casali, Elena Ferrari, Tecla Ciociola, Alberto Spisni, Thelma A. Pertinhez

**Affiliations:** 1 Centro Universitario Odontoiatria, University of Parma, Via Gramsci 14, 43126 Parma, Italy; marco.meleti@unipr.it (M.M.); rita.antonelli@studenti.unipr.it (R.A.); margherita.pezzi@gmail.com (M.E.P.); benedetta.ghezzi@unipr.it (B.G.); mariavittoriaviani@gmail.com (M.V.V.); giacomo.setti@unipr.it (G.S.); 2 Department of Medicine and Surgery, University of Parma, Via Volturno 39, 43125 Parma, Italy; eleonora.quartieri@unipr.it (E.Q.); emanuela.casali@unipr.it (E.C.); tecla.ciociola@unipr.it (T.C.); alberto.spisni@unipr.it (A.S.); thelma.deaguiarpertinhez@unipr.it (T.A.P.); 3 Transfusion Medicine Unit, Azienda USL-IRCCS di Reggio Emilia, Viale Umberto I 50, 43123 Reggio Emilia, Italy

**Keywords:** saliva, metabolomics, salivary gland, parotid, submandibular/sublingual

## Abstract

The detection of salivary molecules associated with pathological and physiological alterations has encouraged the search of novel and non-invasive diagnostic biomarkers for oral health evaluation. While genomic, transcriptomic, and proteomic profiles of human saliva have been reported, its metabolic composition is a topic of research: metabolites in submandibular/sublingual saliva have never been analyzed systematically. In this study, samples of whole, parotid, and submandibular/sublingual saliva from 20 healthy donors, without dental or periodontal diseases, were examined by nuclear magnetic resonance. We identified metabolites which are differently distributed within the three saliva subtypes (54 in whole, 49 in parotid, and 36 in submandibular/sublingual saliva). Principal component analysis revealed a distinct cluster for whole saliva and a partial overlap for parotid and submandibular/sublingual metabolites. We found exclusive metabolites for each subtype: 2-hydroxy-3-methylvalerate, 3-methyl-glutarate, 3-phenylpropionate, 4-hydroxyphenylacetate, 4-hydroxyphenyllactate, galactose, and isocaproate in whole saliva; caprylate and glycolate in submandibular/sublingual saliva; arginine in parotid saliva. Salivary metabolites were classified into standard and non-proteinogenic amino acids and amines; simple carbohydrates; organic acids; bacterial-derived metabolites. The identification of a salivary gland-specific metabolic composition in healthy people provides the basis to invigorate the search for salivary biomarkers associated with oral and systemic diseases.

## 1. Introduction

Human whole saliva (WS) is a mixture of fluids produced by parotid (20%), submandibular (65–70%), sublingual (7% to 8%), minor (<10%) salivary glands, and by gingival sulcus (crevicular fluid) [1]. 

Chemical-physical properties and volume of WS can grossly vary among people, as well as in the same person, according to endogenous and exogenous factors (e.g., age, gender, circadian rhythm, psychological state, nutrition, diseases, drugs, and environmental exposures). Moreover, qualitative variations of saliva, mostly related to the presence and concentration of specific categories of molecules, have been reported [2,3,4].

Variation in salivary flow (e.g., by stimulation with citric acid) is associated with changes in most metabolites’ concentrations. The concentration of acetate in unstimulated saliva is markedly higher than in the stimulated one. By contrast, lactate has more elevated levels in stimulated than in unstimulated saliva. Glucose has a similar concentration in the two types of fluids [5].

It is important to highlight that the inter-individual variability of salivary metabolic profiles seems to be higher than the intra-individual one. Such observation has led to hypothesize that under standardized conditions, an individual metabolic phenotype is relatively stable [2].

Despite the relatively well-described composition of WS, parotid saliva (PS) and submandibular/sublingual saliva (SM/SL), in terms of nucleic acids and proteins [6,7], the metabolites composition of these fluids is still subject of research.

The origin of the metabolites in WS is quite diversified: some molecules are produced by human metabolic processes, others by oral microorganisms, and several are of exogenous origin [2,8,9,10]. Being unlikely a contamination by exogenous and microbial molecules, the majority of the metabolites in PS [5] and SM/SL are presumably host-derived. As for the metabolites of human origin in WS, they either originate within the salivary glands or are released from alive or desquamated oral mucosal cells [8].

To the best of our knowledge, SM/SL metabolites have never been systematically described: only few examples of metabolic profiles have been published so far [11]. In particular, Yamada-Nosaka and co-workers [12] recorded broad and not well resolved proton Nuclear Magnetic Resonance (NMR) spectra of SM/SL, most likely due to the presence of the viscous mucous component.

Currently, the increasing number of studies on salivary metabolites [11] point to interpret the metabolite content of each type of saliva, in light of physiological and pathological changes that characterize each salivary gland.

Indeed, in-depth analysis of the human salivary metabolome may significantly boost: (1) the research of salivary biomarkers for oral and systemic diseases [13,14,15,16] and (2) the interpretation of the metabolic alterations occurring in physiological conditions (e.g., effects of physical exercise, weight changes, activation of specific metabolic pathways) [17,18].

Here, we report and compare the metabolic composition of human unstimulated PS, SM/SL, and WS derived from a cohort of young and healthy volunteers in physiological conditions. Quantitative and qualitative differences between salivary types are discussed, with special emphasis on resident microflora contribution.

## 2. Results

**Figure 1:**
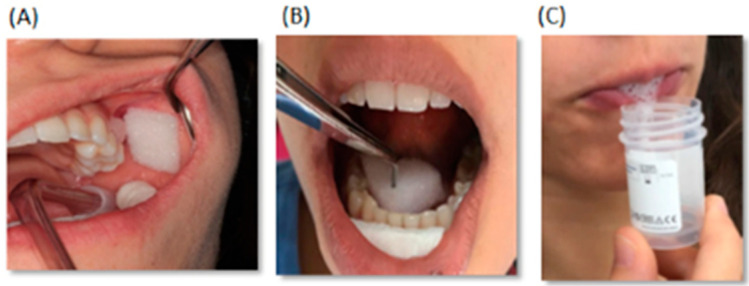
Experimental set-up used for parotid (**A**), submandibular/sublingual (**B**), and whole saliva (**C**) collection.

For each participant (n = 20), a sample of unstimulated PS, SM/SL, and WS was collected separately, in this exact order, as described in the Material and Methods section (Figure 1). 

The salivary flow rates (0.15 ± 0.16 mL/min for PS; 0.20 ± 0.09 mL/min for SM/SL; 0.49 ± 0.28 mL/min for WS, expressed as mean value ± SD) show a wide inter-individual variability, mainly for PS. Our flow rate values of unstimulated saliva are in accordance with flow rates reported in the literature: 0.1, 0.1, and 0.6 mL/min for PS, SM/SL, and WS, respectively [19,20]. Using a sialometry test, we preliminary measured WS flow rate obtaining a good correlation (r = 0.86) with the value measured at the end of the whole sampling procedure. This fact indicates that the flow rate is not significantly altered, even after the prolonged sample collection required for some participants.

### 2.1. Metabolite Content of Saliva Subtypes: Emerging Differences

**Figure 2:**
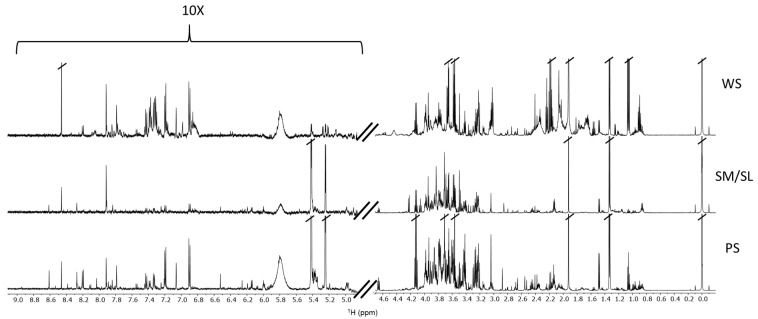
^1^H-NMR spectra of unstimulated WS, PS, and SM/SL saliva from the same participant, acquired at 25 °C. The left region of the spectra shows the vertical scale increased by a factor of 10.

The 1D ^1^H-NMR spectra of WS, SM/SL, and PS samples (Figure 2) highlight different and characteristic metabolites patterns. 

Since the total signal area under each NMR spectrum is proportional to the total metabolite content, the comparison of the values derived from the whole cohort reveals a similar total metabolite content for WS and PS and a sensibly smaller value for SM/SL, with a ratio of 1:1:0.3 (WS:PS:SM/SL). 

**Figure 3:**
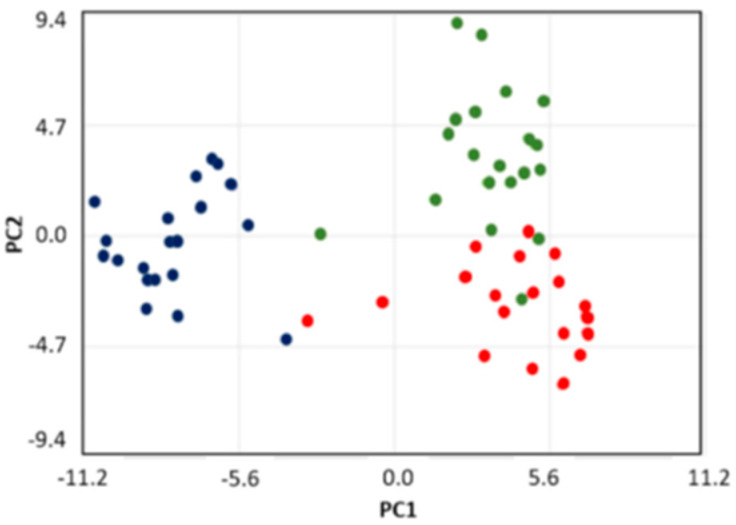
PCA score plot of ^1^H-NMR spectra of whole saliva (blue), parotid saliva (red), and submandibular/sublingual saliva (green) samples. The contribution of the three principal components of the total variance are PC1 = 62.6%, PC2 = 6.8%, and PC3 = 5.1%.

Principal component analysis (PCA) of WS, PS, and SM/SL was applied to binned NMR spectra of all samples (20 samples for each of the 3 salivary types). In Figure 3, WS appears as a well separated cluster of scores, while PS and SM/SL display a partial cluster overlap that might suggest some similarity between their metabolic profiles. As expected, all clusters are characterized by a spread of scores, very likely due to the contribution of inter-individual variability. The principal data variance is given by PC1, and the separation between WS and the other two salivary subtypes is accounted for by lactate, propionate, maltose, 2-aminoadipate, and taurine. 

### 2.2. Salivary Metabolites

**Figure 4:**
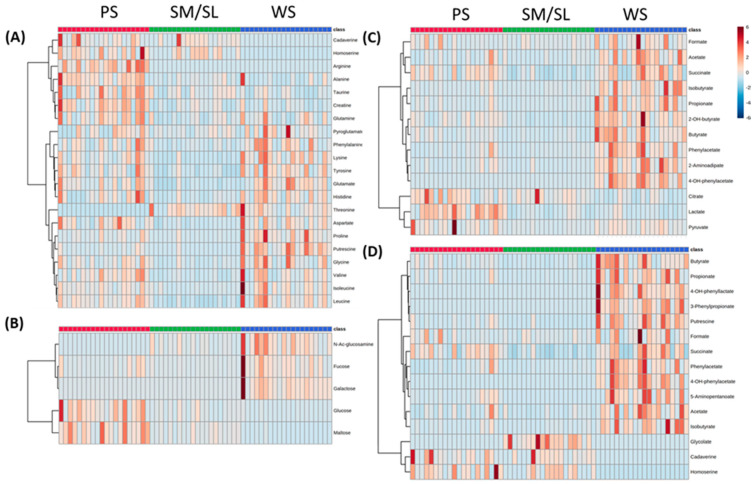
Heatmap analysis of metabolites grouped according to the categories: (**A**) amino acids, (**B**) carbohydrates, (**C**) organic acids, and (**D**) selected prokaryotic metabolites. PS (red), SMS (green), and WS (blue) samples.

We identified 66 metabolites with average concentrations higher than 5 μM. Heatmap analysis of all salivary metabolites profiles highlights the heterogeneity of WS, PS, and SM/SL composition and shows that 54 are in WS, 49 in PS, and 36 in SM/SL (Figure 4). Overall, 32 metabolites (48%) are common to the three saliva subtypes. Notably, it has been possible to single out a number of metabolites uniquely present in each salivary subtype: 2-hydroxy-3-methylvalerate, 3-methyl-glutarate, 3-phenylpropionate, 4-hydroxyphenylacetate, 4-hydroxyphenyllactate, galactose, and isocaproate in WS; arginine in PS; caprylate and glycolate in SM/SL.

### 2.3. Classes of Metabolites

To describe quantitatively each type of saliva, we selected an ensemble of metabolites, focusing on molecules with relevant concentrations and/or differential expression in the three salivary types. The median value and the range of concentration for each of the selected metabolites are reported in Table 1. Table 1—Section A and Figure 4A indicate the presence of the majority of the standard amino acids, non-proteinogenic amino acids, e.g., pyroglutamate and taurine, and biogenic amines, e.g., cadaverine, creatine, homoserine, and putrescine. When the highest median value (Table 1, in bold) is found in WS, most frequently, we observe that the related metabolite concentrations are significantly higher than in SM/SL. Only for lysine, phenylalanine, proline, and putrescine, their WS concentrations are significantly higher also than the ones found for PS. In the case of alanine, creatine, glutamine, and taurine, instead, the PS concentrations result significantly higher than in WS and SM/SL.

In Table 1—Section B, the relevant simple carbohydrates are listed. PS presents high levels of glucose and maltose that are significantly higher than in WS and SM/SL (Figure 4B). 

Finally, Table 1—Section C summarizes the main organic acids. Once more, the highest median value belongs more frequently to WS. Exceptions are lactate and citrate that are significantly higher in PS than in WS and SM/SL (Figure 4C).

Figure 4D and Table 1—Section A and C report the distribution of metabolites that, according to the “Human Metabolome Database” (www.hmbd.ca), are mainly referable to bacterial metabolism. Noteworthy, WS presents high abundance of short chain fatty acids (SCFAs, i.e., formate, acetate, propionate, and butyrate), products of amino acid degradation such as putrescine and 5-aminopentanoate, and metabolic products of aromatic amino acids fermentation such as 4-hydroxy-phenyllactate and 3-phenylpropionate [21]. Yet, some of these metabolites are detected only in gland saliva, possibly reflecting either a microbial contamination or a host gland contribution. In fact, cadaverine and homoserine, detected in PS and SM/SL, are absent in WS; glycolate is detected only in SM/SL.

### 2.4. Salivary Cell Count

**Figure 5:**
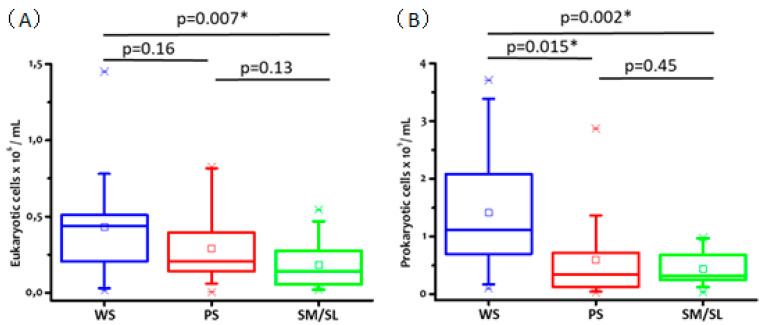
Eukaryotic (**A**) and prokaryotic (**B**) cell count distributions obtained with the three salivary types of all the subjects of the study. The significance level of the independent t-tests is set at 0.05 (* *p* < 0.05).

Prokaryotic cell counts are significantly higher in WS than in PS and SM/SL, reflecting the prokaryotic metabolite proportion observed in the three salivary types (Figure 5). The median value of WS cell distribution (Figure 5) is consistent with the value reported by Sender et al. [22].

In all types of saliva, eukaryotic cell counts range between 0.1 and 0.5 × 10^6^ cells/mL, and they are approximately three orders of magnitude lower than the prokaryotic ones.

## 3. Discussion

Mapping the human oral metabolome, with emphasis on the metabolic composition of saliva subtypes, is expected to provide hints to clarify the physiologic and pathologic processes of the salivary glands and oral cavity. In this study, we were able to identify and quantify a considerably high number of metabolites: 54, 49, and 36 in WS, PS, and SM/SL, respectively. 

Free salivary amino acids are known to be primarily produced by endogenous and exogenous proteases of salivary glands, exfoliating cells, and oral microflora [23]. Proteolytic amino acid-degrading bacteria dissect proteins and peptides into amino acids and convert them in short chain fatty acids [24], contributing, together with the saccharolytic bacteria, to the organic acid content of saliva.

Thus, we hypothesize that the high concentration of amino acids and organic acids found in WS (Table 1—Sections A and C) may reflect bacterial metabolic pathways. 

Indeed, glutamine, glycine, and proline, the most represented residues in salivary Proline-Rich Proteins (PRPs) [25], are among the amino acids found at the highest concentrations (Table 1—Section A) being produced by proteolytic processes. Pyroglutamic acid, the common N-terminal of acidic PRP, is present as well at significant concentrations in saliva samples (Table 1—Section A). Moreover, salivary mucins, a heterogeneous group of glycoproteins synthesized and secreted by the submandibular, sublingual, and minor salivary glands, contribute to proline salivary concentration [26]. Arginine, ornithine, and lysine, which originate from proteins and peptides lysis, are metabolized by oral cavity bacteria, and contribute to the salivary content of putrescine, by decarboxylation of ornithine, an intermediate in the degradation of arginine, and cadaverine, formed by lysine decarboxylation [27]. Our data (Table 1—Section A) point to the presence of cadaverine only in gland salivary types. 

We found a significant concentration of arginine only in PS (Table 1—Section A) in agreement with Van Wuyckhuyse and co-workers [28] that found a concentration of free arginine and lysine in PS of caries-free adults significantly higher than in caries-susceptible individuals. Noteworthy, arginine deiminase system is a relevant source of alkali generation by means of ammonia production. Accordingly, this enzymatic system of both saliva and dental plaque results more active in caries-free people when compared to caries-active individuals, likely contributing to the neutralization of plaque acids and to caries resistance [29]. 

Taurine, a beta-sulfonic amino acid, is probably the most abundant free amino acid in mammalian tissues. As suggested by Revenga-Parra and co-workers [30], the determination of its concentration in various body fluids seems relevant for the early diagnosis of Alzheimer’s disease, growth retardation, diabetes mellitus, epilepsy, sepsis, and some types of cancers. In rat SM glands, taurine is suggested to act as a regulator of the saliva ionic strength [31] and, in human saliva, appears to be correlated to physical stress [18]. Our study shows that such metabolite is present in all three types of saliva with a marked prevalence in PS (Table 1—Section A), thus, suggesting a role in salivary glands function. 

Glucose, a blood component, passes in saliva through the salivary gland apparatus in proportion to its blood concentration. A statistically significant positive correlation has been found between fasting salivary glucose and fasting blood glucose [32]. Our study shows that glucose level in PS not only is considerably higher than in WS, as reported by Wang and co-workers [33], but is also higher than the concentration found in SM/SL, suggesting that parotid gland is the primary route of entry (Table 1—Section B). Therefore, we can assume that, in fasting conditions, such as in our cohort, the WS glucose level is severely influenced by the oral microorganism’s glucose metabolism as well as by fasting blood glucose concentration. 

Salivary glycoproteins are a suite of macromolecules that, while contributing with specific functional roles to the oral cavity defense, constitute endogenous nutrients for the resident oral microflora, thus, being also responsible for the microbial plaque growth [34]. Because glycoproteins degradation is the result of the combined action of various microbial glucosidases [24], we conclude that the prevalent presence of monosaccharides such as fucose, N-acetylglucosamine, and galactose in WS samples (Table 1—Section B) should primarily be associated with the microbial saccharolytic activity on the oligosaccharide chains linked to glycoproteins. 

Interestingly, the unexpected presence of salivary maltose (Table 1—Section B), a significant source of carbohydrate to oral bacteria, might be attributed to the digestive action of α-amylase, an enzyme produced by serous cells of parotid glands, with minor contributions from other glands’ enzymes [35]. On the basis of the results reported here, we can hypothesize that the concentrations of maltose in glandular saliva reflect the concentration of gland α-amylase, while its presence in WS is drastically reduced because of microbial utilization.

Saccharolytic microflora converts sugars to lactic, formic, acetic, succinic, and other organic acids through the glycolytic pathway [24,36]. On the other hand, in subgingival sites, asaccharolytic and/or proteolytic bacteria metabolize nitrogenous compounds derived from gingival crevicular fluid, creating an environment rich in SCFAs and ammonia [24,36]. 

Organic acids, in our saliva samples, are preferentially present in anionic form and frequently display their highest concentrations in WS (Table 1—Section C). 

Consistent with the data reported by Gardner and co-workers [8], we find that acetate is the most abundant metabolite in WS samples, being present at relevant concentrations also in PS and SM/SL saliva. Interestingly, formate, acetate, propionate, and butyrate metabolites are present at a sensibly lower concentration in glandular saliva as compared to WS, a fact that might be due to their reduced bacterial contamination (Figure 5).

Lactate and citrate are more concentrated in PS than in WS and SM/SL, suggesting that the parotid gland is a relevant route of entry of these metabolites into the oral cavity [8]. However, WS lactate concentration may reflect also the contribution of microorganisms and oral mucosa cells [37]. The median lactate concentration that we measured in the WS of individuals with low dental plaque score is in agreement with the value reported by Gardner and colleagues [8].

Eventually, it is worth mentioning that we found a significant concentration of three metabolites of bacterial origin (glycolate, cadaverine, and homoserine) in PS and SM/SL saliva, but not in WS. We interpret the presence of those metabolites as an indication of microbial contamination of saliva. Particularly in the case of the glandular saliva, even if the fluids are collected at the close proximity of the excretory ducts, it is not possible to exclude a bacterial contamination of the outlet of the terminal portion of the salivary ducts, also in the absence of clinical signs of glandular infections. Because the outlets of the Stensen and Wharton ducts are very close to those portions of the dental arches where plaque, calculus, and periodontal diseases are more frequent (vestibular area of maxillary molars and lingual area of mandibular incisors) [38], even following rigorous procedures for saliva collection, it is possible that the area of sponge application is contaminated by dental and periodontal bacterial species. The hypothesis of some bacterial contamination is sustained by the high number of prokaryotic cells measured in all types of saliva (Figure 5). The absence of those metabolites in WS, on the other hand, may be the result of a pronounced dilution effect and/or additional degradation processes.

Overall, we have been able to identify and evaluate the concentration of a relevant number of metabolites in human saliva and to highlight qualitative and quantitative differences between WS, PS, and SM/SL saliva. Particularly, for the first time, we provided a metabolic profile of SM/SL.

We believe that mapping the human salivary metabolome is central for understanding most of the physiologic and pathologic oral metabolic pathways, including those related to the host–microbiome relationships.

## 4. Materials and Methods

The present study was approved by the Ethical Committee of the “Area Vasta Emilia Nord” (AVEN) (protocol number: 808/2018/SPER/UNIPR METASAL3). Written consent was obtained from all volunteers who participated in this study, according to the Declaration of Helsinki. 

### 4.1. Subjects of the Study

A cohort of twenty healthy volunteers (10 males, 10 females), aged 19–25 years, qualified for saliva collection after oral clinical examination, interview for data acquisition on general medical history, and salivary flow rate assessment by sialometry test (modified Saxon Test) [39]. None of them revealed a full-mouth plaque score (FMPS) and/or full-mouth bleeding score (FMBS) higher than 25%.

### 4.2. Saliva Collection 

For each participant, a sample of PS, SM/SL, and WS was collected separately and in the absence of stimulation. Participants were asked to refrain from eating, smoking, and performing intense physical activity for at least 12 h before salivary sampling and to drink only water. Furthermore, it was requested not to carry out oral hygiene (tooth brushing and flossing) in the 45 min before saliva collection. The procedure took place between 8:00 a.m. and 10:00 a.m. to minimize the influence of the circadian rhythm on salivary composition. Immediately before collection, patients rinsed their mouth with water for 1 min. For PS and SM/SL collection, the outlets of the Stensen and Wharton ducts were isolated and gently cleaned with a sterile gauze. A sterile sponge, capable of absorbing saliva flow, was positioned on the outlet of the ducts. Periodically, the sponge was squeezed and a syringe was used to collect the saliva within a vial. WS was collected by the passive drooling method (Figure 1).

During collection, salivary samples were transferred to a tube containing NaN_3_ (0.5% final concentration) and kept on ice until a volume of 5.4 mL, of each salivary type, was obtained and then frozen at −80 °C. 

### 4.3. Sample Preparation and ^1^H-NMR Spectra Collection and Analysis

Each frozen saliva sample was thawed at room temperature and centrifuged at 15,000× *g* for 10 min at 4 °C to remove eukaryotic and prokaryotic cells, cellular debris, and mucins, according to Gardner et al. [40]. The supernatants were protein-depleted by ultra-filtration, using Amicon Ultra-4 Centrifugal filters (3000 MWCO, Merck Millipore) at 4000× *g* for 120 min at 10 °C, and lyophilized.

For ^1^H-NMR measurements, each of the lyophilized samples was suspended in potassium phosphate buffer (50 mM, pH 7.4) and 3-trimethylsilyl propanoic acid (TSP) was added as the chemical shift reference (0.00 ppm) and quantitative internal standard.

High-resolution one-dimensional (1D) ^1^H-NMR spectra acquisition and processing were carried out according to Pertinhez et al. [41]. Metabolites identification and quantification were carried out using Chenomx NMR Suite 8.3 software (Chenomx Inc., Edmonton, AL, Canada).

Heatmap analysis was carried out on targeted metabolites, with concentrations higher than 5 μM at least for one saliva subtype. Heatmaps were generated using MetaboAnalystR (https://www.metaboanalyst.ca) [42], with normalization referenced to TSP and autoscaling. 

### 4.4. Cell Counting

Eukaryotic cells (oral epithelial cells and leucocytes) and prokaryotic cells were counted according to Gardner et al. [40], to estimate their possible contribution to the metabolic profile of WS, SM/SL, and PS. 

### 4.5. Statistical Analysis

To compare the metabolite composition of each saliva subtype, the upper-tailed Mann–Whitney test (Origin 2019 software) was applied. P < 0.05 was considered statistically significant. The saliva subtype with the highest median concentration of each metabolite is shown in bold in Table 1. 

Unsupervised multivariate analysis. To produce an overview of the overall variability, NMR spectra datasets were analyzed by principal component analysis (PCA), using the PCA module of MestreNova 11.0 software (Mestrelab Research, Santiago de Compostela, Spain). 

## Figures and Tables

**Table 1:** Concentrations of selected metabolites in whole, parotid, and submandibular/sublingual saliva.

METABOLITE ^a^	WS (μM)	PS (μM)	SM/SL (μM)
**SECTION A: STANDARD AND NON–PROTEINOGENIC AMINO ACIDS**			
**Alanine**	27.4 (9.4–212.6)	69.1 (41.5–219.1) ° ^§^	20.7 (8.1–74.9)
**Arginine**	ND	24.2 (5.9–56.3)	ND
**Aspartate**	19.4 (9.8–76.7)	25.7 (7.9–64.0) ^§^	7.3 (1.8–18.7)
**Cadaverine ^b^**	ND	3.2 (0.7–17.6)	2.5 (0–16.6)
**Creatine**	10.8 (6.7–49.4)	48.9 (23.7–116.7) ° ^§^	16.6 (6.4–48.1)
**Glutamate**	108.1 (30.6–250.1) ^§^	57.5 (27.7–224.0)	19.4 (8.6– 71.2)
**Glutamine**	28.4 (6.6–134.6)	63.3 (9.1–151.4) ° ^§^	21.6 (2.3–70.6)
**Glycine**	82.5 (12.1–306.6) ^§^	66.6 (3.6–192.3)	14.4 (4.5–76.5)
**Histidine**	20.1 (4.1–49.9) ^§^	17.6 (7.8–70.7)	5.2 (2.9–21.1)
**Homoserine ^b^**	ND	36.4 (0–158.4)	32.4 (3.2–57.9)
**Isoleucine**	5.0 (0.9–44.3) ^§^	3.8 (2.0–13.4)	1.2 (0.7–4.1)
**Leucine**	13.2 (3.5–56.9) ^§^	12.0 (6.3–30.7)	4.8 (2.1–9.5)
**Lysine**	66.7 (17.6–164.8) * ^§^	19.4 (10.1–97.5)	6.2 (2.2–37.9)
**Phenylalanine**	16.8 (6.4–48.6) ^§^ *	10.9 (5.0–41.1)	4.6 (2.0–12.7)
**Proline**	64.1 (24.8–446.8) * ^§^	41.1 (10.8–156.0)	7.2 (2.8–74.4)
**Putrescine** **^b^**	38.6 (8.5–96.4) * ^§^	5.1 (0.6–27.3)	0.9 (0.5–17.7)
**Pyroglutamate**	12.9 (2.9–70.5) ^§^	9.4 (0–32.3)	7.6 (3.3–14.7)
**Taurine**	46.2 (2.8–132.0)	121.4 (0–342.2) ° ^§^	60.2 (3.3–146.9)
**Threonine**	4.6 (2.4–31.4)	ND	7.3 (2.9–22.3)
**Tyrosine**	34.9 (10.5–93.5) ^§^	28.2 (14.38–90.9)	10.1 (3.0–30.8)
**Valine**	9.3 (2.9–59.2)	12.4 (3.0–38.0) ^§^	4.7 (1.1–13.1)
**SECTION B: SIMPLE CARBOHYDRATES**			
**Fucose**	34.8 (11.5–275.8) * ^§^	5.6 (0.9–57.9)	4.8 (2.4–16.3)
**Galactose**	18.9 (6.3–173.9)	ND	ND
**Glucose**	11.8 (6.8–137.8)	204.6 (81.8–697.8) ° ^§^	46.8 (7.6–211.8)
**Maltose**	1.3 (0.2–52.8)	296.9 (103.5–1587.9) ° ^§^	76.5 (16.4–420.4)
**N-acetylglucosamine**	26.3 (2.2–141.5) ^§^	ND	10.5 (1.4–40.8)
**SECTION C: ORGANIC ACIDS**			
**2-Aminoadipate**	186.0 (77.2–530.3) * ^§^	25.0 (2.4–117.3)	11.6 (2.0–92.8)
**2-Hydroxybutyrate**	13.6 (1.4–53.1) * ^§^	7.6 (4.3–12.8)	2.5 (0.9–6.5)
**3-Phenylpropionate ^b^**	10.0 (2.0–38.1)	ND	ND
**4-hydroxyphenylacetate ^b^**	8.6 (1.9–19.0)	ND	ND
**4-hydroxyphenyllactate ^b^**	4.4 (0.7–23.9)	ND	ND
**5-Aminopentanoate ^b^**	100.5 (0–386.3) *	12.1 (0–82.2)	ND
**Acetate ^b^**	2277.9 (734.1–4322.8) * ^§^	470.7 (81.8–3145.0)	237.4 (54.0–1370.4)
**Butyrate ^b^**	20.9 (3.2–77.3) * ^§^	5.1 (0.8–14.8)	4.0 (1.1–18.6)
**Citrate**	12.7 (0.7–33.0)	35.3 (16.0–125.8) ° ^§^	20.4 (5.9–146.9)
**Formate ^b^**	37.7 (8.7–234.0) * ^§^	15.1 (6.8–106.7)	17.2 (6.5–97.9)
**Glycolate ^b^**	ND	ND	7.8 (1.8–55.4)
**Isobutyrate ^b^**	18.6 (3.76–47.6) * ^§^	1.3 (0.2–9.9)	1.7 (0.3–5.2)
**Lactate**	123.1 (23.7–517.5)	714.8 (408.8–1683.9) ° ^§^	162.7 (84.0–444.5)
**Phenylacetate ^b^**	15.9 (4.2–46.2) * ^§^	1.3 (0–25.7)	1.6 (0.3–3.3)
**Propionate ^b^**	261.8 (64.7–627.9) * ^§^	31.2 (4.4–200.8)	17.5 (4.3–151.7)
**Pyruvate**	18.8 (4.0–52.8) ^§^	12.1 (1.4–232.0)	5.1 (2.1–14.2)
**Succinate ^b^**	16.1 (9.9–39.2) * ^§^	12.1 (3.8–23.3)	5.1 (2.2–11.8)

^a^ In each section, metabolites are presented in alphanumerical order. The values reported for each metabolite are median concentrations in WS, PS and SM/SL. Numbers in brackets indicate the concentration range. Numbers in bold are the highest median values obtained in the three saliva types; For each metabolite, a Mann–Whitney test has been applied for comparing the concentration pool producing the highest median value with that of the other saliva samples, when detected. The significance level has been set at 0.05; The symbols °, *, and ^§^ are associated with the highest median values and indicate that the related metabolite concentration tends to be significantly higher than in WS, PS, or SMS, respectively; ^b^ Metabolite of bacterial origin, according to the “Human Metabolome Database” (www.hmbd.ca); ND is the abbreviation for not detected.
